# The Impact of Heat Stress and Water Deficit on the Photosynthetic and Stomatal Physiology of Olive (*Olea europaea* L.)—A Case Study of the 2017 Heat Wave

**DOI:** 10.3390/plants7040076

**Published:** 2018-09-20

**Authors:** Matthew Haworth, Giovanni Marino, Cecilia Brunetti, Dilek Killi, Anna De Carlo, Mauro Centritto

**Affiliations:** 1 Tree and Timber Institute, National Research Council of Italy (CNR-IVALSA), Via Madonna del Piano 10, 50019 Firenze, Italy; marino@ivalsa.cnr.it (G.M.); brunetti@ivalsa.cnr.it (C.B.); decarlo@ivalsa.cnr.it (A.D.C.); mauro.centritto@cnr.it (M.C.); 2 Department of Agrifood Production and Environmental Sciences (DiSPAA), University of Florence, Viale delle Idee 30, 50019 Firenze, Italy; 3 Institute of Biometeorology, National Research Council of Italy (CNR-IBIMET), Via Giovanni Caproni 8, 50145 Firenze, Italy; dilek.killi@gmail.com

**Keywords:** heat stress, drought, water use efficiency, OJIP, stomatal conductance, Mediterranean agriculture

## Abstract

Heat waves are predicted to increase in frequency and duration in many regions as global temperatures rise. These transient increases in temperature above normal average values will have pronounced impacts upon the photosynthetic and stomatal physiology of plants. During the summer of 2017, much of the Mediterranean experienced a severe heat wave. Here, we report photosynthetic leaf gas exchange and chlorophyll fluorescence parameters of olive (*Olea europaea* cv. Leccino) grown under water deficit and full irrigation over the course of the heat wave as midday temperatures rose over 40 °C in Central Italy. Heat stress induced a decline in the photosynthetic capacity of the olives consistent with reduced ribulose-1,5-bisphosphate carboxylase/oxygenase (RubisCO) activity. Damage to photosystem II was more apparent in plants subject to water deficit. In contrast to previous studies, higher temperatures induced reductions in stomatal conductance. Heat stress adversely affected the carbon efficiency of olive. The selection of olive varieties with enhanced tolerance to heat stress and/or strategies to mitigate the impact of higher temperatures will become increasingly important in developing sustainable agriculture in the Mediterranean as global temperatures rise.

## 1. Introduction

As mean global temperatures rise, the frequency of more extreme climatic events such as droughts and heat waves will increase [1]. This will have severe consequences for agriculture in areas such as the Mediterranean that are presently characterized by hot dry summers, where vegetation experiences high evapotranspirative demand and photo-oxidative stress. Olive (*Olea europaea* L.) is cultivated across the Mediterranean and is the basis for agro-industrial products worth more than €11 billion each year [2]. In late July to August 2017, much of Europe experienced a pronounced heat wave resulting in extensive forest die-back and loss of crops [3]. Analysis of the physiological response of olive to extreme climatic events will elucidate the likely impacts of future climatic change.

Drought events are often associated with transient anomalous increases in temperature above normal levels known as heat waves. However, the effects of drought and heat stress on plant physiology are often considered in isolation in experimental studies. Both drought and heat stress adversely affect the carbon and water efficiency of plants [4,5,6], and as such their combined effects on plant physiology warrant greater attention. As the availability of water within the soil declines, free ABA in the leaf rises inducing stomatal closure to reduce stomatal conductance (*G*_s H2O_) and transpirative water-loss [7,8,9]. During drought, the amount of energy utilized for photochemistry declines [10], and if not dissipated safely via protective processes such as non-photochemical quenching [11], this excess energy may induce oxidative stress through the production of reactive oxygen species [12]. The thylakoid membranes of the chloroplast are particularly sensitive to oxidative stress; an effect apparent in reduced photosystem II (PSII) electron transport when assessed using chlorophyll fluorescence (ChlF) [13,14]. The conductance of CO_2_ across the mesophyll layer (*G*_m CO2_) also frequently declines under drought stress [4,15,16] due to stomatal closure reducing CO_2_ uptake, lower CO_2_ assimilation within the chloroplast and biochemical changes in the mesophyll layer that reduce transport of CO_2_ [17,18,19]. Elevated temperature exacerbates the effects of drought by reducing the ratio of photosynthesis relative to respiration ratio, and as a consequence the carbon balance. Moreover, heat stress increases the soil water content threshold (i.e., the amount of water that the plant can extract) at which point plant functions decrease [20].

The thylakoid membranes are also extremely sensitive to heat stress [21]. However, the accumulation of heat shock proteins can act to stabilize the thylakoid membranes during exposure to high temperature [22]. Heat stress can also affect photosynthetic CO_2_ assimilation via reduced specificity of ribulose-1,5-bisphosphate carboxylase/oxygenase (RubisCO) for CO_2_ (i.e., an increase in rates of photorespiration relative to photosynthesis (*P*_N_)), lower solubility of CO_2_ and reduced activity of RubisCO activase [23,24,25,26]. The lower photochemistry associated with reduced RubisCO activity during heat stress [25] would also reduce the capacity of photosystem I to act as an electron receiver from PSII, potentially exacerbating the negative impact of high temperature on PSII [27]. Heat stress may also adversely affect plant water relations by increasing *G*_s H2O_ [28,29]. However, longer-term stomatal adaptation to growth at higher temperatures (as oppose to instantaneous increases in leaf temperature) may result in no increase in *G*_s H2O_ [20,27,30,31]. More detailed analysis of the photosynthetic and stomatal responses to temperature of plants from contrasting environments would enable a greater understanding of the likely impacts of heat waves on different vegetation types.

Combined drought and heat wave events are likely to have an increasing influence on the productivity of agricultural and natural vegetation [32]. Through analysis of the photosynthetic responses of olive grown under full irrigation and water deficit during the heat wave of summer 2017, we aimed to: (i) assess the impact of heat stress on the carbon and water efficiencies of olive; (ii) quantify the impact of heat stress on the biochemical and diffusive constraints to photosynthetic CO_2_ uptake; (iii) investigate the interaction of heat stress with plant water status and their impact on photosynthetic performance; and (iv) discuss the likely impact of future increased drought and heat wave events on the viability of olive production in the Mediterranean region.

## 2. Results

**Figure 1:**
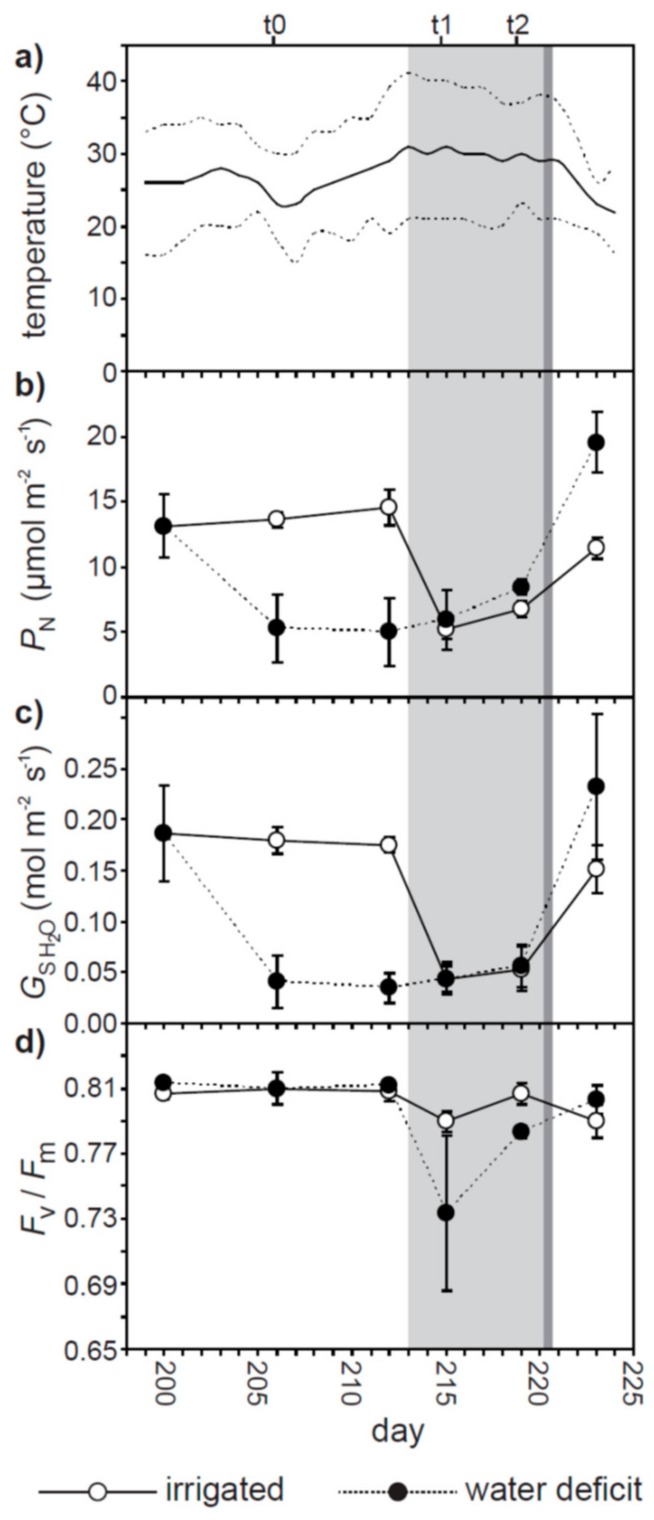
(**a**) Daily mean (solid black line), maximum and minimum (dashed lines either side of the mean) temperature during the experiment coinciding with the heat wave of summer 2017 (marked in light grey shading) which occurred from day 213 to 221. The darker grey shading marks the point at which the water deficit plants were ‘re-watered’ to receive full irrigation. Point measurements of photosynthesis (*P*_N_) (**b**), stomatal conductance (*G*_s H2O_) (**c**) and the maximum quantum efficiency of PSII (*F*_v_/*F*_m_) (**d**) of olive plants subject to full irrigation (white fill symbols, solid line) and water deficit (black fill symbols, broken line) were recorded at intervals during the experimental treatment. Error bars indicate one standard deviation either side of the mean. Time periods t0 (day 206), t1 (day 215) and t2 (day 219) marked above panel a refer to OJIP analysis in Figure 4.

From 25 to 31 of July 2017 (day 206 to 212) mean daily temperatures in Sesto Fiorentino rose from 23 to 32 °C. During this period, the maximum daily temperature increased from 31 to 41 °C (Figure 1a). This increase in daily average and maximum temperatures coincided with respective 63.9 and 75.4% reductions of *P*_N_ and *G*_s H2O_ in olive plants receiving full irrigation (Figure 1b,c). The effect of higher temperatures was less apparent on the already low values of *P*_N_ and *G*_s H2O_ observed in olive subject to water deficit. The maximum quantum efficiency of PSII (*F*_v_/*F*_m_) of both the well-watered and water deficit olive plants declined respectively by 2.2 and 9.7% from t0 to t1 as the heat wave developed (Figure 1d). A significant difference in *F*_v_/*F*_m_ values between the irrigated and water deficit plants was only observed at t2 (day 219) during the heat wave (one-way ANOVA F_1,8_ = 9.800; *P* = 0.035).

**Figure 2:**
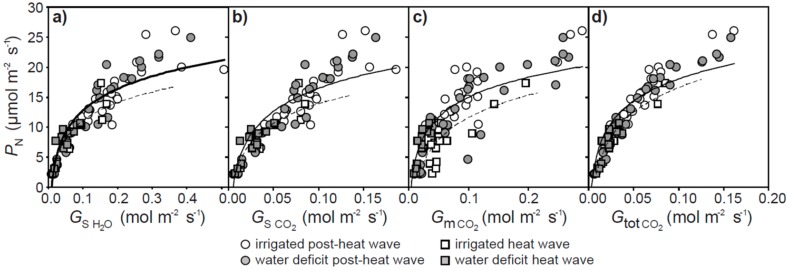
The relationship between photosynthesis (*P*_N_) and stomatal conductance to water vapor (*G*_s H2O_) (**a**), and stomatal (*G*_s CO2_) (**b**), mesophyll (*G*_m CO2_) (**c**) and total (*G*_tot CO2_), (**d**) conductance to CO_2_ of olive plants during (square symbols, dashed best fit line) and after (circle symbols, solid best fit line) the heat wave (marked in light grey shading in Figure 1) subject to full irrigation (white fill symbols) and water deficit (grey fill symbols) treatment.

**Figure 3:**
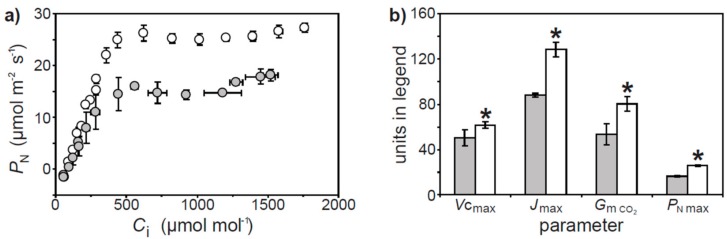
The response of photosynthesis (*P*_N_) to increasing [CO_2_] within the internal leaf air-space (*C*_i_) (**a**) and physiological parameters calculated from the *P*_N_-*C*_i_ response curve utilizing the method of Ethier and Livingston [33] (**b**) in well-watered olive plants during (grey fill symbols) and after (white fill symbols) the heat wave. Y-axis units for *V*c_max_, *J*_max_, and *P*_N max_ are μmol m^−2^ s^−1^, and *G*_m CO2_ is measured in mmol m^−2^ s^−1^. * indicates significant difference between measurements conducted during and after the heat wave using a one-way ANOVA: *V*c_max_ (F_1,7_ = 11.0; *P* = 0.013), *J*_max_ (F_1,7_ = 199.1; *P* = 2.1 × 10^−6^), *G*_m CO2_ (F_1,7_ = 7.3; *P* = 0.031) and *P*_N max_ (F_1,7_ = 151.1; *P* = 1.8 × 10^−5^). Error bars indicate one standard deviation either side of the mean.

Instantaneous measurements of leaf gas exchange indicate that the rate of *P*_N_ was positively related to *G*_s H2O_, *G*_s CO2_, *G*_m CO2_, and *G*_tot CO2_ prior to and during the heat wave (Figure 2). However, the relationships between *P*_N_ and diffusive conductances to CO_2_ became less steep during the heat wave. The lower *P*_N_ observed during the heat wave may have been associated with biochemical impairment of CO_2_ assimilation (Figure 3a). Analysis of the response of *P*_N_ to *C*_i_ in well-watered plants during and after the heat wave indicated that *V*c_max_ and *J*_max_ were reduced by 17.6 and 31.6% respectively. The maximum rate of *P*_N_ was 36.3% lower in well-watered plants assessed during the heat wave. The conductance of CO_2_ across the mesophyll layer when assessed from the *P*_N_-*C*_i_ curve utilizing the curve fitting method of Ethier and Livingston [33] was 33.3% lower during the heat wave (Figure 3b). A similar 36.4% reduction was observed in *G*_m CO2_ values calculated using the variable *J* method of olive plants receiving full irrigation during the heat wave (Figure 2c).

**Figure 4:**
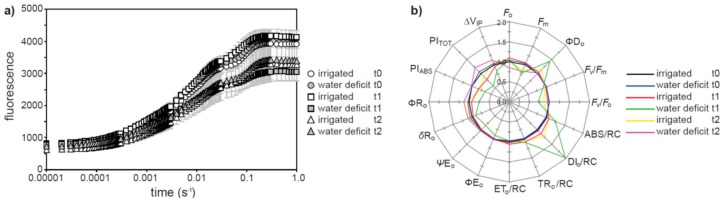
Analysis of the chlorophyll fluorescence transient of olive plants at t_0_ (circle symbol), t_1_ (square symbol) and t_2_ (triangle symbol) (see Figure 1 for sampling intervals) subject to full irrigation (white fill symbols) and water deficit (grey fill symbols) treatments: (**a**) average OJIP induction curves; (**b**) spider plot of parameters (see Materials and Methods for definitions and descriptions) extrapolated from the OJIP transient expressed in relation to values of plants receiving full irrigation at t_0_. Error bars indicate one standard deviation either side of the mean.

Analysis of the OJIP transient of chlorophyll a fluorescence suggests that photochemical PSII electron transport was reduced in both irrigated and water deficit olive plants during the heat wave (Figure 4a). The impact of the heat wave on PSII was most apparent in the olive plants grown under water deficit at t_1_, where the quantum yield of energy dissipated (ΦD_o_) and the flux of energy dissipated for each reaction center (DI_o_/RC) were respectively 42.9% and 97.4% greater than well-watered plants at t_0_ (Figure 4b). The fluorescence maximum (*F*_m_) (−22.0%), the activity of the oxygen evolving complex on the donor side of PSII (*F*_v_/*F*_o_) (−28.6%), the use of harvested excitation energy for electron transport to the primary plastoquinone A acceptor of PSII (ΨE_o_) (−16.6%), the quantum yield of the reduction of the final stage acceptors at the PSI stage (δR_o_) (−24.4%), the efficiency of the electron chain flux in the I to P phase (∆V_IP_) (−16.9%) and photochemical and non-photochemical energy absorption of both chlorophyll antennae (PI_ABS_) (−33.6%) and PSII reaction centers (PI_TOT_) (−43.8%) were all reduced in olive plants grown under water deficit during the initial stages of the heat wave at t_1_. Heat stress also induced respective reductions of 17.4% and 21.0% in PI_ABS_ and PI_TOT_ values of olives grown under full irrigation at t_1_. The impact of heat stress on OJIP parameters was less apparent at t_2_ than t_1_ in both the well-watered and water deficit treated olive plants (Figure 4b).

**Figure 5:**
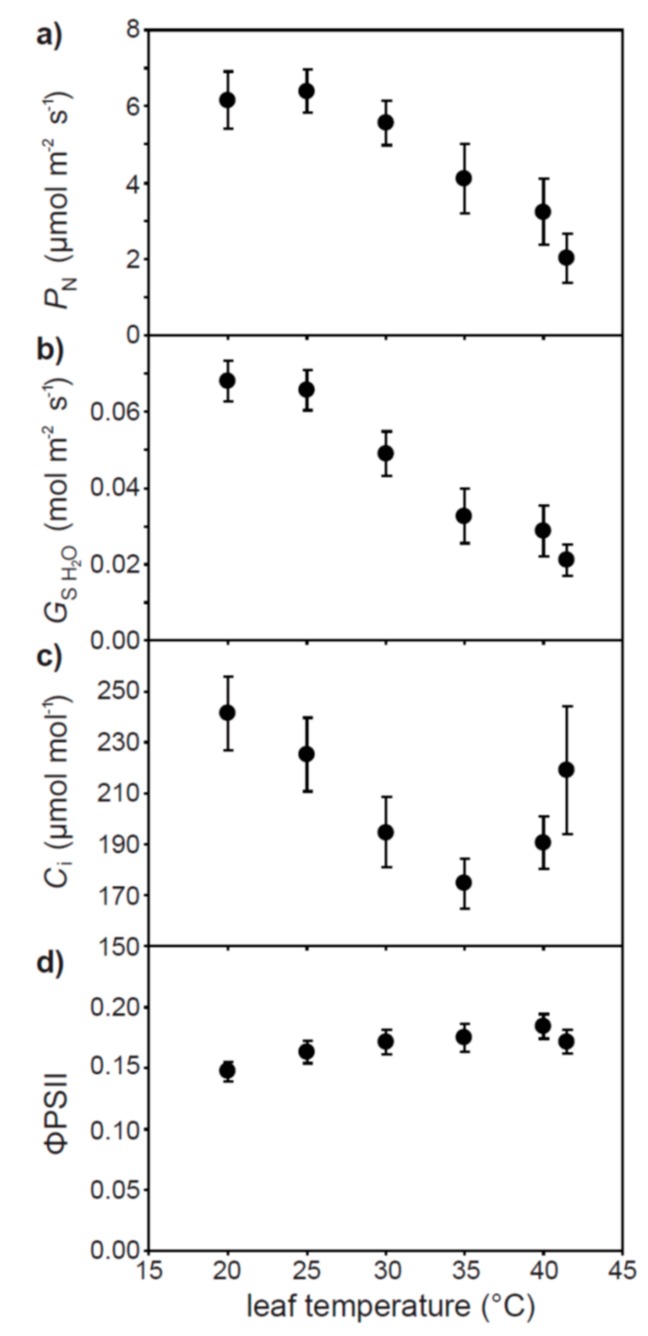
Response of photosynthesis (*P*_N_) (**a**), stomatal conductance (*G*_s H2O_) (**b**), intercellular sub-stomatal air-space [CO_2_] (*C*_i_) (**c**), and the actual quantum efficiency of PSII under steady state conditions in the light (ΦPSII) (**d**) to an instantaneous increase in leaf temperature over a range of 20 to 41.5 °C of well-watered olive plants. Error bars indicate one standard deviation either side of the mean.

Instantaneous increases in leaf temperature from 20 to 41.5 °C resulted in respective 67.0% and 69.0% reductions in *P*_N_ (Figure 5a) and *G*_s H2O_ (Figure 5b). The sub-stomatal concentration of CO_2_ declined by 27.8% as leaf temperature rose from 20 to 35 °C, before increasing to 219.1 μmol mol^−1^ [CO_2_] at a leaf temperature of 41.5 °C (Figure 5c). The actual quantum efficiency of PSII in the light adapted state (ΦPSII) remained relatively constant between 0.147 to 0.184 at all leaf temperatures (Figure 5d).

## 3. Discussion

Olive is highly adapted to environments characterized by low water availability and high evapotranspirative demand [16,34,35,36]. However, the results of this study suggest that the significant heat wave experienced during summer 2017 had a pronounced detrimental impact on the photosynthetic physiology of olive plants subject to both well-watered and water deficit treatments. This interaction between heat stress and plant water status is likely to play an increasingly prominent role in the productivity of olive trees as heat waves and droughts become more prevalent in the Mediterranean. Transcriptome analysis suggests that the genetic expression pathways involved in plant responses to drought and heat stress are largely distinct, despite the common coincidence of these abiotic stresses [37]. Our findings would suggest that the impact of heat stress on olive varies depending upon the water status of the plant.

Photosynthesis declined in well-watered olive plants both during the heat wave (Figure 1b) and when exposed to instantaneous increases in leaf temperature (Figure 5a). The retention of ΦPSII in the well-watered olive plants exposed to an instantaneous increase in leaf temperature (Figure 5d) would indicate that in this instance the reduction in *P*_N_ is largely the result of a corresponding increase in photorespiration [23]. However, the well-watered olive plants exposed to the heat wave exhibited reductions in PI_ABS_ and PI_TOT_ (Figure 4b), indicative of lower photochemistry [14]. This is consistent with reductions in the photosynthetic capacity of the well-watered olive plants during the heat wave (Figure 3b). Similar reductions in *V*c_max_ and *J*_max_ have also been observed in crop [27] and tree [20,38] species when grown at higher temperatures, and is likely the result of reductions in RubisCO activase [25]. It was not possible to fully remove diffusive limitations from the olive plants grown under water deficit cf. [39] during the heat wave to accurately assess photosynthetic capacity [40]. It is noteworthy that instantaneous gas exchange measurements of *P*_N_ were not further reduced during the heat wave in the plants subject to water deficit (Figure 1b). Analysis of the ChlF OJIP transient indicated that PSII was more strongly impaired, with more energy dissipated per reaction center, in the water deficit plants than in their well-watered counterparts. Indeed the reduced capacity for photochemical energy usage in the water deficit plants may have exacerbated the deleterious impact of heat stress on the thylakoid membranes of the olives e.g., [6]. Lower ΨE_o_, ∆V_IP_, ΦR_o_, and δR_o_ in the water deficit olive plants during heat stress would be consistent with reduced plastoquinone A to B electron transport and PSI electron acceptors (e.g., Figure 3b in the well-watered plants) [41,42]. This disruption to intersystem electron transport and PSI end electron acceptor associated with heat stress likely resulted in the generation of reactive oxygen species exacerbating the oxidative stress experienced by the thylakoid membranes of water deficit plants [43,44]. The absence of any reduction in *P*_N_ in the water deficit olive plants during the heat wave may suggest that *P*_N_ was largely determined by diffusive rather than biochemical constraints e.g., [45,46,47].

Previous studies have observed increases in *G*_s H2O_ associated with higher temperatures in herbaceous plants [28,48,49] and woody trees [29,50,51]. As temperatures rise, the increase in transpirative cooling associated with higher *G*_s H2O_ may serve to prevent leaf temperatures reaching harmful levels [52]. In contrast, we observed a reduction in *G*_s H2O_ in olive during both the heat wave (Figure 1c) and, similar to results obtained in adult olive trees growing in field conditions [53], when exposed to an instantaneous increase in leaf temperature within the leaf cuvette (Figure 5b). Longer-term adaptation to temperature likely affects the response of *G*_s H2O_ to instantaneous variations in leaf temperature [27,30,31,54]. An increase in leaf to air vapor pressure deficit (VPD) with temperature [50] may have induced stomatal closure in the well-watered olive plants through ABA synthesis [55]. Higher leaf to air VPD induces reduced *G*_s H2O_ [8], and this likely played a role in the physiological response of the plants as the maximum daily temperature rose from 35 to ~40 °C (Figure 1a). However, leaf to air VPD was maintained constant throughout the assessment of the impact of instantaneous increases in leaf temperature on leaf gas exchange (Figure 5b); therefore, the results of the present study may suggest that short and longer-term stomatal responses to higher temperatures may be species specific when considered in the context of contrasting short and long term stomatal responses to heat stress e.g., [27,28,31]. The reduction in *G*_s H2O_ observed in the well-watered olives during the heat wave (Figure 1c) and instantaneous increases in leaf temperature (Figure 5b) may reflect an adaptation to minimize the risk of xylem embolism e.g., [32,56,57]. Selective pressures exerted by growth in an environment characterized by low water availability and high evapotranspirative demand may favor a reduction in *G*_s H2O_ with temperature (rather than the positive relationship between *G*_s H2O_ and temperature reported in other studies: [28,29]), as higher transpiration rates would result in lower xylem vessel pressures potentially leading to cavitation e.g., [58]. It is noteworthy that the higher temperatures associated with the heat wave did not induce further reductions in the *G*_s H2O_ values of olive plants subject to water deficit treatment. Stomatal conductance of the well-watered and water deficit treated olive plants was identical at t1 and t2 (Figure 1c), raising the possibility that this represents the limit of stomatal closure e.g., [59,60] in this variety of olive.

Heat stress associated with the heat wave generally impaired photosynthetic CO_2_ transport (Figure 2) [21]. Similar reductions in the relationship between *P*_N_ and CO_2_ uptake were observed in C3 species grown at high temperatures (but not plants with C4 photosynthesis, where CO_2_ is concentrated within the bundle sheath to minimize the impact of the reduced CO_2_-specificity of RubisCO) [27,30]. The conductance of CO_2_ across the mesophyll layer was reduced during the heat wave (Figure 2c and Figure 3b). This was likely associated with greater biochemical limitations to assimilation of CO_2_ (Figure 3a) and stomatal closure (Figure 1c and Figure 2a) reducing the flux of CO_2_ across the mesophyll [18]. The reduction in the relationship between *P*_N_ and total conductance to CO_2_ may reflect an increase in photorespiration relative to *P*_N_ [23] and non-photochemical energy usage (Figure 4b) in the olive plants subject to heat stress [61].

The results of this study indicate that heat stress will result in impaired photosynthetic carbon gain in olives as heat waves increase in frequency, duration and severity. In contrast to other studies, heat stress did not adversely affect the water balance of olive leaves, as *G*_s H2O_ declined as temperatures rose. Nonetheless, damage to PSII was more apparent in olive plants subject to water deficit (Figure 4). As the yield of olive fruit is closely related to water availability during fruit development (specifically: flower formation, flowering/fruit set, and rapid fruit growth) [34,62], exposure to heat stress during this period would likely have further negative impacts on productivity by exacerbating any pre-existing diffusive and biochemical limitations to *P*_N_ e.g., [63]. Physiological analysis of the impact of drought and heat stress provides a valuable insight into the photosynthetic and stomatal adaptation of olive to growth under conditions characterized by low water availability, elevated temperatures, and high evapotranspirative demands. Treatments such as the application of kaolin to olive trees (which covers the leaf surface in clay particles)—e.g., [64]—may have additional benefits in reducing energy interception of the leaf by increasing surface albedo, and could be an effective mitigation strategy to reduce leaf thermal stress during heat waves. Phenotyping studies to identify olive varieties with attributes conducive to tolerance of heat and drought stress may also be effective in ensuring the sustainability of olive production in the Mediterranean.

## 4. Materials and Methods

### 4.1. Plant Material and Growth Conditions

Ten two-year-old olive (*Olea europaea* L. var. Leccino) plants were potted in 10 dm^3^ pots filled with sand. The plants were grown outside for two months prior to the experiment in full sunlight in Sesto Fiorentino, Central Italy, and watered each day to pot capacity and supplied each week with 100 mL full strength Hoagland nutrient solution (equivalent to an electrical conductivity of 2.0 dS m^−1^) to provide nutrients at free access rates [65]. The evening prior to the instigation of the water deficit treatment on day 200 (19 July 2017), the plants were watered to pot water capacity. The pot capacity (PC) water content was determined gravimetrically. The pots were weighed each day and the amount of water lost via evapotranspiration was replaced after the well-watered (80% of PC) and water deficit (when plants had reached 20% of the starting *G*_s H2O_ values, this was approximately 30% of PC) weight targets had been achieved in five replicate plants for each water treatment. On the evening of day 220 (8 August 2017), the plants subject to the water deficit treatment were ‘re-watered’ to 80% PC and this pot water content was maintained for the remainder of the experiment. The minimum, maximum, and mean daily temperatures were recorded by a nearby weather station (~100 m from the experimental site) managed by the Institute of Biometeorology of the National Research Council of Italy.

### 4.2. Leaf Gas Exchange Analysis

Point measurements of leaf gas exchange and ChlF were performed on the uppermost fully expanded leaf of each replicate plant (five replicates for each water treatment) between 09:00 a.m. and 11:00 a.m. using a LiCor Li6400XT fitted with a 6400-40 2 cm^2^ leaf cuvette (Li-Cor, Inc., Lincoln, NE, USA). Conditions in the leaf cuvette were set to a photosynthetic photon flux density (PPFD) of 2000 μmol m^−2^ s^−1^, leaf temperature of 30 °C, [CO_2_] of 400 μmol mol^−1^ and relative humidity of 60%. The multi-phase fluorescence setting was used with an initial saturating pulse of 8000 μmol m^−2^ s^−1^ [66]. The quantum efficiency of PSII under steady state conditions in the light (ΦPSII) was determined following Genty et al. [67]. Mesophyll conductance (*G*_mCO2_) was calculated using the variable J method described by Harley et al. [68]. Total conductance to CO_2_ (*G*_totCO2_) was calculated as [4]

$$G_{\mathrm{tot}\mathrm{CO}2}=\frac{G_{S\mathrm{CO}2}*G_{m\mathrm{CO}2}}{G_{S\mathrm{CO}2}+G_{m\mathrm{CO}2}}$$

The response of *P*_N_ to increasing [CO_2_] within the internal sub-stomatal air-space (*C*_i_) was determined during the heat wave on day 216 (4 August 2017) and after the heatwave on day 226 (14 August 2017) on well-watered olive plants using a LiCor Li6400-40 attached to a 6 cm^2^ LiCor 6400-02B leaf cuvette. To remove stomatal limitations to *P*_N_, the concentration of [CO_2_] within the leaf cuvette was lowered to 50 μmol mol^−1^ for 60 min to fully open stomata (thus removing any diffusive limitations to *P*_N_: [39]) before [CO_2_] was increased in stages when after 3 to 4 min *P*_N_ had stabilized ([CO_2_] steps: 50, 100, 200, 300, 400, 600, 800, 1000, 1200, 1400, 1600, 1800, 2000 μmol mol^−1^). Leaf temperature was 25 °C and relative humidity 60% throughout the *P*_N_-*C*_i_ response curve. Exhaust air from the LiCor Li6400 was fed into an air-space between the leaf gasket and a supplementary external gasket to reduce the impact of diffusive leaks [69]. The maximum carboxylation rate of RubisCO (*V*c_max_), the maximum rate of electron transport for regeneration of ribulose-1,5-bisphosphate (RuBP) (*J*_max_), and *G*_m CO2_ were calculated from the *P*_N_-*C*_i_ curves following Ethier and Livingston [33]. The maximum rate of *P*_N_ (*P*_Nmax_) was considered to be *P*_N_ at a PPFD of 2000 μmol m^−2^ s^−1^ and [CO_2_] of 2000 μmol mol^−1^. To assess the impact of instantaneous increases in leaf temperature on leaf gas exchange parameters, the uppermost leaf from five well-watered plants was assessed using a LiCor Li6400XT and 6400-40 2 cm^2^ leaf cuvette fitted with a 6400-88 Expanded Temperature Kit that allows hot/cold water to cool/heat the Peltier thermoelectric blocks in the cuvette. A Thermo Fisher Haake A28 (Thermo Fisher Scientific, Waltham, MA, USA) water bath was used to pass water through the blocks adjacent to the Peltiers. Following Bunce [28], the water passing through the blocks was below that of the desired leaf temperature, so that the Li6400 was always in the heating mode. Leaf gas exchange and ChlF parameters were recorded at leaf temperatures of 20, 25, 30, 35, 40, and 41.5 °C (this was the maximum leaf temperature achievable with this system). Conditions in the leaf cuvette were: PPFD of 1000 μmol m^−2^ s^−1^, [CO_2_] of 400 μmol mol^−1^ and leaf to air vapor pressure deficit was maintained constant at 2.0 ± 0.2 KPa by altering the amount of vapor within the reference gas stream entering the leaf cuvette.

### 4.3. Chlorophyll Fluorescence

Chlorophyll fluorescence analyses were performed between the hours of 11:00 a.m. and 12:00 p.m. on the same leaves used in the leaf gas exchange analyses. Transient analysis of chlorophyll a fluorescence was undertaken using a Hansatech Pocket-PEA (plant efficiency analyser) fluorimeter (Hansatech, King’s Lynn, UK). Leaves were dark adapted for 30 min and then exposed to a saturating light pulse (intensity >3000 μmol m^−2^ s^−1^, excitation light of 650 nm) [70]. This results in a polyphasic transient of chlorophyll fluorescence: O (20–50 µs), J (2 ms), I (30 ms), and P (peak). The theoretical basis and analysis of OJIP curves is given in Strasser et al. [70]. The OJIP curves were analyzed using Biolyzer 4 HP v.3 (Bioenergetics Laboratory, University of Geneva, Switzerland). The parameters extrapolated from the OJIP curve and analyzed in this study are listed and defined in Appendix A.

## Appendix A

Parameters calculated from the ChlF OJIP transient from Strasser [70].

*F*_o_	minimum fluorescence yield in dark-adapted conditions.
*F*_m_	maximum fluorescence yield in dark-adapted conditions.
ΦD_o_	quantum yield of energy dissipation.
*F*_v_/*F*_m_	maximum quantum yield of PSII photochemistry.
*F*_v_/*F*_o_	an indicator of the activity of the oxygen evolving complex on the donor side of PSII.
ABS/RC	absorption of chlorophyll antennae per reaction center.
DI_o_/RC	flux of energy dissipated for each reaction center.
TR_o_/RC	flux of trapped energy per reaction center leading to the reduction of plastoquinone A.
ET_o_/RC	electron flux beyond plastoquinone A per reaction center.
ΦE_o_	initial quantum yield of electron transport.
ΨE_o_	probability that harvested excitation energy is utilized for electron transport to the primary plastoquinone A acceptor of PSII.
*δ*R_o_	efficiency of electron carriers in reducing end electron acceptors at the PSI acceptor.
ΦR_o_	quantum yield of the reduction of final stage acceptors at the PSI stage.
PI_ABS_	a performance index based on the photochemical and non-photochemical energy absorption of chlorophyll antennae.
PI_TOT_	performance index incorporating the concentration of reaction centers.
ΔV_IP_	efficiency of the electron chain flux in the I to P phase of the chlorophyll a fluorescence.
